# Simulation of the Mesoscale Cracking Processes in Concrete Under Tensile Stress by Discrete Element Method

**DOI:** 10.3390/ma18132981

**Published:** 2025-06-24

**Authors:** Zhenyu Zhu, Bintang Mas Mediamartha, Shuyang Yu, Yifei Li, Jian Xu, Pingping Gu

**Affiliations:** 1 School of Transportation and Civil Engineering, Nantong University, Nantong 226019, China; zhuzhenyu@stmail.ntu.edu.cn (Z.Z.); mmpbintang@gmail.com (B.M.M.); 2 Department of Mechanical Engineering, Huzhou University, Huzhou 313002, China; yifeili286@gmail.com; 3 Power China Kunming Engineering Corporation Limited, Huzhou 313002, China; xujian7897@163.com

**Keywords:** mesoscale concrete, Discrete Element Method (DEM) simulation, crack propagation, mesoscopic behavior of concrete

## Abstract

Material scientists face a critical challenge in characterizing the mesoscopic damage evolution of concrete under tensile loading, as traditional experimental and theoretical approaches struggle to resolve the complexities of its multiphase heterogeneous structure. This study addresses this gap by employing the Discrete Element Method (DEM) with PFC2D to model concrete’s mesoscopic cracking, integrating aggregates, mortar, interfacial transition zones (ITZ), and pores. Through parameter calibration against experimental data, uniaxial tensile simulations reveal how aggregate percentages (30–45%) and pore percentages (1–6%) influence crack propagation and tensile strength. Specifically, when the aggregate percentage increased from 30% to 40%, the peak tensile strength decreased by 26%, while increasing from 40% to 45% led to a recovery in strength. With porosity increasing from 2% to 4%, the peak strength dropped by approximately 3%, and further to 6% caused a 14% reduction, demonstrating the quantitative impact of microstructural parameters on concrete performance. Simulation results align closely with experimental data, validating DEM’s efficacy in modeling mesoscopic cracking. This work provides a mesoscopic theoretical foundation for optimizing concrete’s tensile properties and underscores the need to incorporate realistic mesoscopic features in future simulations.

## 1. Introduction

As one of the most widely used materials in modern civil engineering, the mechanical properties and durability of concrete are directly related to the safety and service life of major infrastructure such as bridges, high-rise buildings, and nuclear power facilities [1,2,3,4,5]. However, due to the multiphase heterogeneity of its internal structure, concrete is prone to mesoscopic cracking under external loads, temperature fluctuations, and environmental degradation, which can lead to mechanical deterioration and even structural failure [6,7,8,9,10]. For example [11], the Malpasset arch dam in France collapsed in 1954 due to cracking within the concrete at the dam foundation, which rendered the structure unable to withstand external pressure. Similarly, in 2019, a dam in Jharkhand, India, developed cracks that eventually led to failure under the combined effects of complex topographical conditions, temperature differentials between the interior and exterior of the dam body, and external loading. Therefore, uncovering the evolution mechanisms of mesoscopic cracking in concrete under tensile loading is critical for overcoming the current limitations in optimizing its tensile performance and ensuring the long-term safety of major engineering structures.

Experimental studies offer a valuable means to reveal the changes in the mechanical properties of concrete and provide deeper insight into the factors that may affect its structural stability. In order to quantitatively investigate the failure mechanisms of concrete under external loading, researchers have conducted extensive experiments to uncover the deterioration patterns of mechanical properties and the mechanisms of crack initiation, thereby contributing to improved engineering design and disaster prevention [12,13,14]. For example, Yu et al. [15] employed acoustic emission (AE) techniques to study the effect of different water–cement ratios on the strength of concrete specimens under uniaxial compression; Yang et al. [16] investigated the strength variation of wet-mix shotcrete under different loading rates; Liu et al. [17] examined the influence of varying proportions of crushable coarse aggregates on the mechanical behavior of concrete; Li et al. [18] conducted uniaxial compression tests to explore the effects of aggregate size on concrete strength, and the results showed that the peak strength increased with larger aggregate diameters. However, the tensile strength of concrete is only about 1/20 to 1/10 of its compressive strength, making it more susceptible to crack initiation and failure due to minor defects under tensile loading. Therefore, the failure mechanisms of concrete under tensile loading are equally worthy of investigation [19]: Naseri et al. [20] studied the effects of specimen size and shape on the tensile strength of concrete; Zhao et al. [21] explored changes in tensile strength and energy dissipation of concrete after high-temperature exposure; Chen et al. [22] found that the tensile strength of concrete increases with the strength of the interfacial transition zone (ITZ). Nguyen et al. [23] investigated the early-age tensile behavior of concrete and discovered that its tensile strength increases rapidly, approximately four hours after initial mixing, providing insights for concrete mix design and curing practices. These diverse experimental designs have significantly advanced the understanding of concrete’s tensile behavior; however, experimental approaches are typically limited to macro-level observations and offer only restricted insight into the microscopic evolution of damage and failure mechanisms.

Theoretical studies, through mathematical modeling and mechanical principles, refine patterns derived from experimental observations and enable a deeper understanding of the failure mechanisms of concrete [24]. Žarković et al. [25] developed a local analysis model for concrete based on the coupling of plasticity theory and damage mechanics; Wang et al. [26] proposed a damage model for concrete under complex stress conditions based on the equivalence of damage energy release; Miehe et al. [27] introduced a dissipative crack surface density function within the diffusive crack theory framework and proposed an incremental variational formulation to describe crack propagation in concrete; Zhao et al. [28] developed a staggered elastoplastic phase-field model based on the Drucker–Prager criterion to investigate the macroscopic nonlinear mechanical behavior of concrete; Wu et al. [29] proposed a plastic damage constitutive model for concrete based on damage energy release rate, which not only reproduces the mechanical responses observed in experiments but also provides predictions of damage distribution. However, theoretical models face challenges in describing multiscale behavior and often adopt simplified boundary conditions, limiting their ability to capture the complex crack propagation and mechanical response of heterogeneous concrete structures.

With the advancement of science and technology, numerical simulation has also made significant progress. Numerical simulation overcomes the limitations of traditional experimental and theoretical studies, such as poor repeatability, high cost, and low time efficiency. It also provides an effective approach for visualizing and quantifying meso-scale mechanisms, thus enabling a more comprehensive understanding of concrete failure processes [30,31,32,33]. Li et al. [34] investigated the effects of porosity and crack density on the effective elastic modulus of concrete using FEM; Yu et al. [35] analyzed the influence of random aggregate size and distribution on the mechanical properties of concrete via FEM; Wu et al. [36] simulated concrete tensile tests using FEM and found that the strength measured from splitting tension tests was 5% to 70% higher than that obtained from direct tensile tests; Li et al. [37] investigated the influence of cracks on concrete performance using FEM. The results showed that the tensile strength decreased inversely with crack width when the width was less than 0.55 mm, while it remained stable for widths greater than 0.55 mm. As one of the most widely used numerical methods, FEM has contributed significantly to the study of concrete mechanical behavior. However, it is computationally expensive and limited by mesh discretization when simulating crack initiation and propagation. The discrete element method (DEM) effectively addresses these issues by directly simulating the microstructure of materials, making it more suitable for investigating damage mechanisms [38,39]. Nitka et al. [40] simulated the failure of concrete under three-point bending using DEM and monitored the associated acoustic emission characteristics; Yang et al. [41] investigated the failure modes and mechanical behavior of concrete under different aggregate distributions and ITZ strengths using DEM; Zhao et al. [42] simulated the fracture behavior of concrete using DEM and found that the presence of mortar could suppress crack propagation. DEM has made notable progress in simulating crack propagation and deformation characteristics of concrete, providing valuable insights for enhancing engineering safety. However, studies focusing on the tensile behavior of concrete using DEM remain limited, and understanding fracture mechanisms across different concrete components remains a critical research topic.

In summary, while existing studies have advanced the understanding of concrete tensile behavior, critical gaps remain: (1) Most DEM simulations focus on compressive failure rather than tensile loading, limiting insights into crack initiation mechanisms under tensile stress; (2) FEM may be limited in simulating crack propagation; (3) The quantitative impact of aggregate gradation and porosity on mesoscopic crack propagation paths lacks systematic analysis; (4) Few studies integrate realistic microstructural features to establish a “composition-mechanics” correlation. This study addresses these limitations by employing DEM to simulate tensile failure processes, aiming to reveal the mesoscopic damage evolution mechanisms and provide a theoretical basis for optimizing concrete tensile performance through microstructural parameter control.

## 2. Materials and Methods

**Figure 1:**
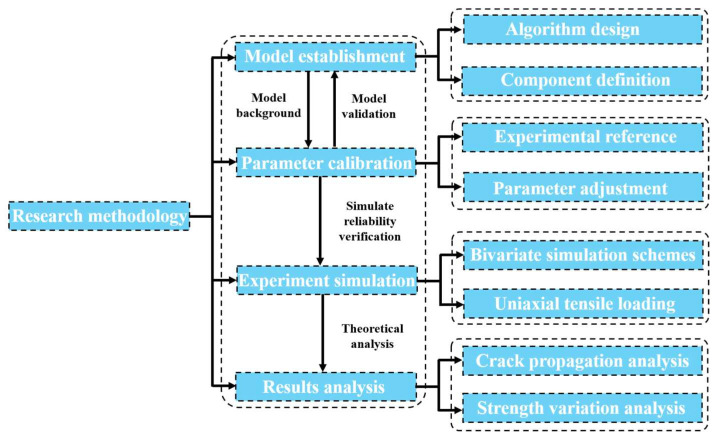
Flowchart of methodology.

The methodology flowchart of this study is shown in Figure 1. By combining numerical simulation and theoretical analysis, the mechanical properties and structural degradation of concrete under tensile load were revealed under different aggregate percentages and porosities.

### 2.1. DEM (Discrete Element Method) Modeling

The multiphase heterogeneous structure of concrete and its discontinuous characteristics during failure determine the limitations of traditional continuum models. DEM, through discrete particle modeling and contact mechanics analysis, can not only naturally capture the initiation and propagation of cracks, but also quantitatively reveal the influence of micro parameters such as aggregates and pores on macroscopic properties. In this study, numerical simulations were conducted using the discrete element method (DEM) software PFC2D5.0. In such software, the physical model is composed of circular particles, and the equilibrium and displacement between discrete particles are solved by tracking the motion of individual particles. PFC2D employs simplified constitutive models to represent the complex internal mechanisms of the physical system. Once calibrated under a specific stress state, the constitutive parameters can be applied to other stress states, enabling the simulation of models under various loading conditions.

**Figure 2:**
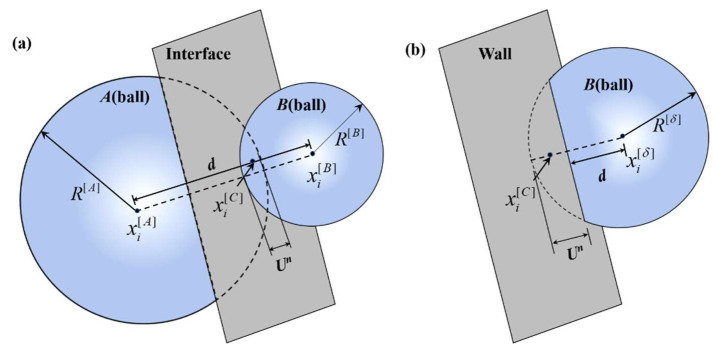
Contact elements in PFC2D. (**a**) Contact between particles and (**b**) contact between particles and walls.

The force-displacement law serves as the fundamental basis for the simulation. As shown in Figure 2, the contact forces and displacements between particles are calculated at the contact points within their overlapping regions. The calculation formula for the unit normal vector of the contact surface between two particles is given as follows:

(1) $$n_{i}=\frac{x_{i}^{\left[B\right]}-x_{i}^{\left[A\right]}}{d}$$

where $x_{i}^{\left[A\right]}$ and $x_{i}^{\left[B\right]}$ represent the positions of the centers of adjacent particles *A* and *B*, respectively, and *d* represents the distance between the centers of the particles.

The calculation formula for the overlap between particles is as follows:

(2) $$U^{n}=\left\{\begin{array}{l} R^{\left[A\right]}+R^{\left[B\right]}-d(ball-ball)\\ R^{\left[\delta \right]}-d(ball-wall) \end{array}\right.$$

where $R^{\left[A\right]}$ and $R^{\left[B\right]}$ represent the radius of particles *A* and *B*, respectively. The normal contact force $F^{n}$ transmitted between the particles can be calculated as follows:

(3) $$F^{n}=K^{n}U^{n}n_{i}$$

where $K^{n}$ is the normal contact stiffness coefficient. The shear contact force is related to the loading process, path, and particle motion, so it is recorded in the form of increments. The total shear force is zero when the contact begins to form, and the shear force caused by the relative displacement of each time step will be accumulated to the current value:

(4) $$\Delta F^{s}=-k^{s}\Delta U^{s}$$

where $k^{s}$ is the tangential contact stiffness coefficient and $\Delta U^{s}$ is the tangential relative displacement increment.

### 2.2. Materials

**Figure 3:**
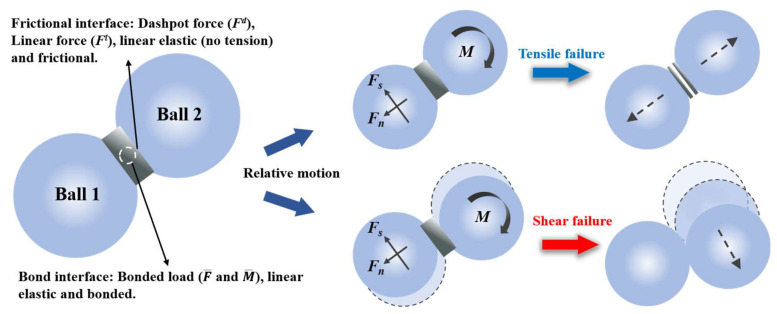
Rules of PBM.

There are multiple model selections built into PFC2D. This paper uses the parallel bond model (PBM) to build the model, and its principle is shown in Figure 3. PBM provides two contact interfaces, namely the friction interface that can transmit damping force and linear force, and the bond section that can transmit bond force and moment. The calculation formulas for the total contact force and moment are as follows:

(5) $$F_{c}=F^{l}+F^{d}+\bar{F}$$

(6) $$M_{c}=\bar{M}$$

where $F^{l}$ is the linear force, $F^{d}$ is the damping force, $\bar{F}$ is the bonding force, and $\bar{M}$ is the bending moment of the bonding contact. As the external force increases, the particles move relative to each other. When the tensile or shear stress at any point exceeds its ultimate strength, corresponding damage will occur.

### 2.3. Model Establishment

As a multiphase composite material, concrete consists of the following mesoscopic components: mortar, aggregates, the interfacial transition zone (ITZ), and pores. Mortar is composed of cement hydration products and unhydrated particles; it encases the aggregates, hardens to form the matrix, and serves as the primary medium for stress transfer. Aggregates, comprising sand and gravel of various sizes, account for approximately 40–50% of the concrete volume. They are randomly distributed and possess high mechanical strength, serving as the primary load-bearing framework of the material. The ITZ is a thin interfacial region between the aggregate and mortar, characterized by high porosity and low mechanical strength, making it the weakest component in concrete. Pores are formed due to water evaporation or external loading and can significantly affect the strength of concrete.

**Figure 4:**
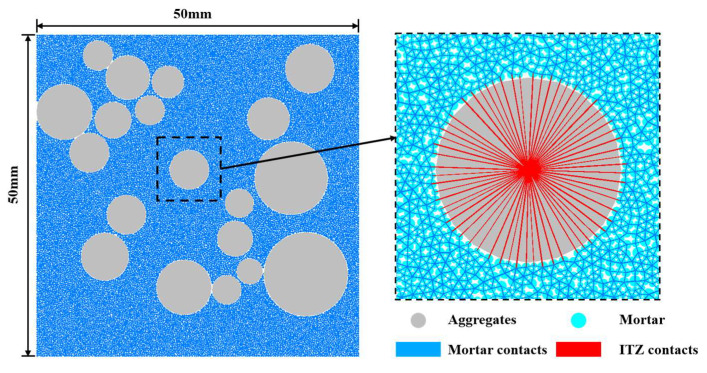
Microstructure of concrete.

The mesoscopic concrete model, illustrated in Figure 4, consists of three material types and their corresponding contacts: mortar, aggregates, and the ITZ between aggregates and mortar. In the model, aggregates and mortar are represented by particles of different sizes distributed randomly. Pores are generated by specifying a porosity level, and the ITZ is defined by modifying the contact properties between mortar and aggregate particles.

**Figure 5:**
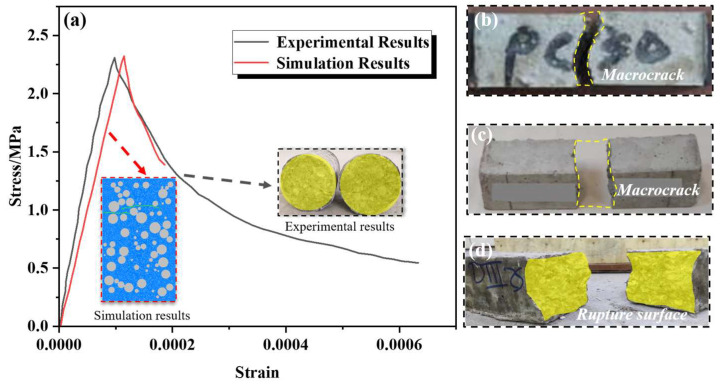
Results of parameter calibration and comparison of failure modes. (**a**) Comparison of experimental and simulation results [40]; (**b**) tensile failure mode of concrete [44]; (**c**) tensile failure mode of concrete [20]; (**d**) tensile failure mode of concrete [45].

Based on the microstructure described above, parameter calibration was first carried out. The calibration was performed using previous experimental results as the reference, in which the specimen was a cylinder with a diameter of 240 mm and a height of 400 mm [43]. Loading was applied by imposing relative motion on selected particles at the top and bottom of the specimen, with a tensile loading rate of 5 mm/s. A two-dimensional model of identical dimensions was constructed in this study. By iteratively adjusting the mesoscopic parameters, the simulation results were made to closely match the experimental outcomes. The final results, shown in Figure 5, indicate that the simulated stress–strain curve closely resembles the experimental curve, with comparable peak strength. In addition, the failure modes are consistent, exhibiting tensile failure along a horizontal cross-section. The failure occurred exclusively within the mortar phase, while the aggregates remained intact. This confirms that the calibrated mesoscopic parameters can be applied to the simulation of other specimens. The specific mesoscopic parameters are listed in the following Table 1.

## 3. Simulation Results

### 3.1. Simulation Scheme Settings

To investigate the fracture mechanisms of concrete under tensile loading with varying aggregate and pore percentages, two sets of simulation schemes were established: (1) Aggregate percentage variation schemes: A-1: *P_agg_* = 30%; A-2: *P_agg_* = 35%; A-3: *P_agg_* = 40%; A-4: *P_agg_* = 45%. (2) Pore percentage variation schemes: B-1: *P_pore_* = 1%; B-2: *P_pore_* = 2%; B-3: *P_pore_* = 4%; B-4: *P_pore_* = 6%. To ensure the reliability of the simulation results, a controlled variable strategy was adopted for the two scheme groups. In Scheme A, the pore percentage was kept constant at 2%. While in Scheme B, the aggregate percentage was fixed at 35%. The aggregate particle size ranged from 3 mm to 20 mm, while the mortar particle size ranged from 0.1 mm to 0.2 mm.

### 3.2. Failure Process and Morphology

**Figure 6:**
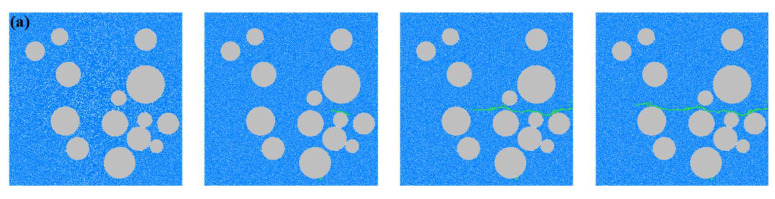

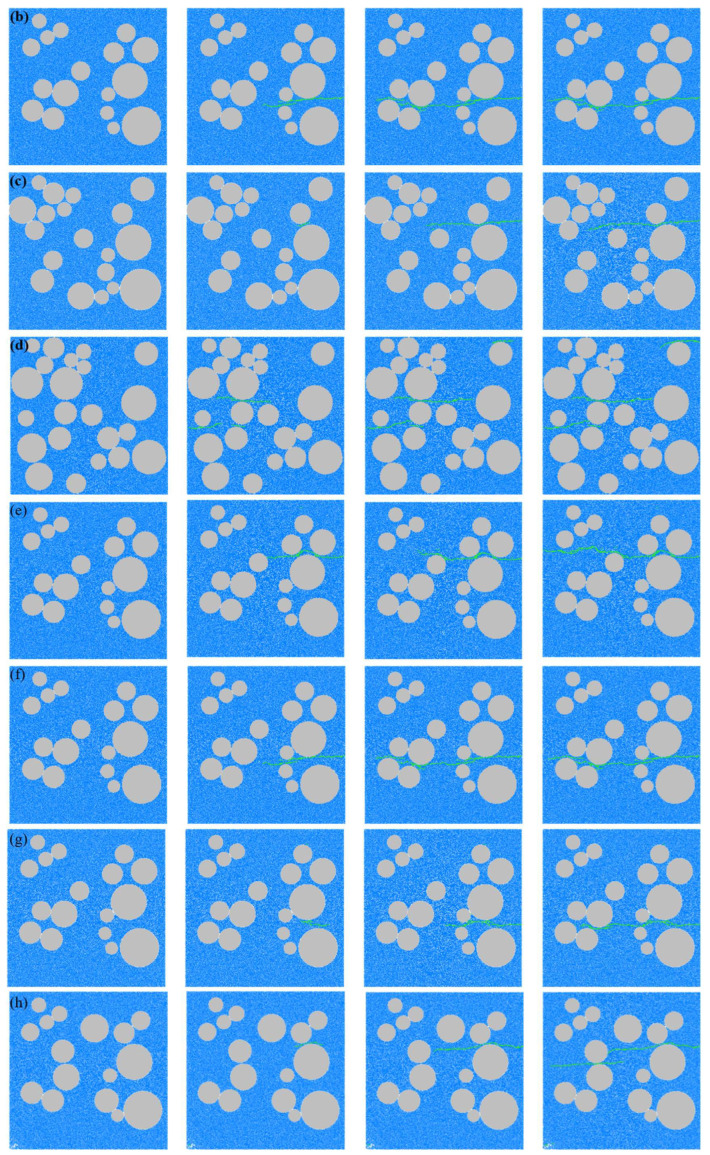
Crack growth in concrete under different schemes. (**a**) A-1: *P_agg_* = 30%; (**b**) A-2: *P_agg_* = 35%; (**c**) A-3: *P_agg_* = 40%; (**d**) A-4: *P_agg_* = 45%; (**e**) B-1: *P_pore_* = 1%; (**f**) B-2: *P_pore_* = 2%; (**g**) B-3: *P_pore_* = 4%; (**h**) B-4: *P_pore_* = 6%.

Figure 6 illustrates the crack evolution and final failure patterns of concrete under different simulation schemes. In all cases, a transverse macroscopic fracture band is observed, characterized by damage propagation along the aggregates, which is consistent with experimental observations. For schemes with varying aggregate percentages, when the aggregate percentage is *P_agg_* = 30%, the sparse distribution of aggregates results in a relatively large mortar volume. Cracks initiate at stress concentration zones within the mortar, such as pores or weak particle contacts, and propagate rapidly along weak interfaces, forming pronounced straight-line initial cracks. As the aggregate percentage increases to 35%, the aggregates begin to impede crack propagation. Cracks tend to bypass the high-strength aggregate particles and propagate along the ITZ, resulting in a more severe failure mode. With a further increase in *P_agg_*, the aggregate distribution becomes denser, significantly increasing the ITZ area, and cracks continue to propagate predominantly along the ITZ. When *P_agg_* reaches 45%, crack propagation frequently encounters aggregate particles, leading to an increased number of cracks and more complex crack patterns. The final failure is characterized by multiple microcracks coalescing through the ITZ and mortar matrix, forming a dominant transverse fracture, while the aggregate particles remain intact due to their higher strength. The results indicate that increasing the aggregate percentage leads to more tortuous crack paths and enhanced energy dissipation capacity.

For schemes with varying pore percentages, at *P_pore_* = 1% and 2%, the pores serve as limited initial defects due to their low density. Cracks primarily initiate at weak particle contacts within the mortar or ITZ, showing relatively regular propagation paths and failure modes similar to dense specimens. When the pore percentage increases to 4%, the pore density becomes significantly higher, and cracks preferentially initiate at the edges of well-connected pores. Due to stress concentration around these pores, cracks rapidly propagate toward aggregate clusters, forming localized multi-crack convergence zones and eventually a transverse fracture band. As the pore percentage further increases to 6%, pores dominate the crack propagation process. The influence of ITZ on crack orientation weakens, and cracks rapidly connect through the mortar, exhibiting more diverse crack patterns and forming compound failure modes.

The aggregates, as the high-strength phase in concrete, act as physical barriers that alter crack propagation paths, forcing cracks to extend along the ITZ rather than through the aggregate particles. Pores, as structural defects, reduce the local effective load-bearing area and serve as crack initiation and propagation sites. At high porosity levels, the internal continuity of concrete is compromised, making it easier for cracks to connect through pores and resulting in a low energy dissipation and rapid failure mode.

### 3.3. Variation in Tensile Strength

**Figure 7:**
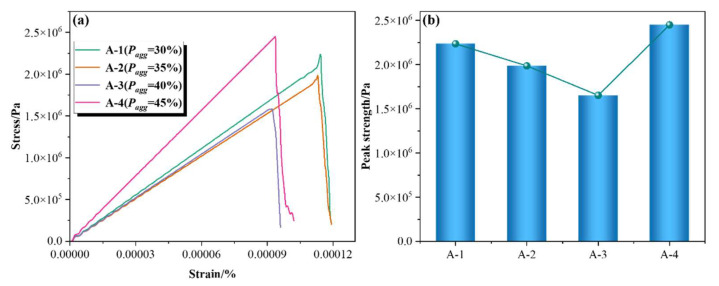
Variation of tensile strength of concrete with different aggregate percentages. (**a**) Stress–strain curve and (**b**) variation of peak strength.

Figure 7 illustrates the variation in the tensile strength of concrete with different aggregate percentages. It can be observed that the stress–strain curves under tensile loading are similar to those under compressive loading, both exhibiting four distinct stages: (1) Elastic stage: The stress–strain relationship is linear, indicating no material damage. All components jointly contribute to load bearing; the ITZ and mortar withstand tensile stress, while aggregates serve as a structural skeleton to transfer the load. (2) Crack initiation stage: When the stress reaches a certain threshold, microcracks begin to appear at weak points within the ITZ or mortar. This leads to a deviation from the linear behavior in the stress–strain curve due to a reduction in stiffness. (3) Crack propagation stage: Microcracks gradually expand and coalesce to form a dominant crack. The stress may reach a peak and subsequently decrease, accompanied by increased energy dissipation. (4) Failure stage: The main crack penetrates the specimen, resulting in a sharp drop in stress and complete loss of load-bearing capacity. The fracture surface typically develops within the mortar or ITZ.

Regarding schemes with different aggregate percentages, the peak tensile strength exhibits a trend of decreasing first and then increasing with the rise in aggregate percentage. When the aggregate percentage *P_agg_* increases from 30% to 40%, the peak strength decreases by 26%. This decline is attributed to the simultaneous expansion of the ITZ area as the aggregate percentage rises. Given that the ITZ is a weaker component in the microstructure with lower mechanical strength than the mortar matrix, it becomes a preferential path for crack initiation and propagation, thereby reducing the overall tensile strength. As *P_agg_* further increases from 40% to 45%, the peak strength recovers and reaches its maximum. This recovery is attributed to the skeleton effect of aggregates, where the enhanced structural support at high percentages significantly improves the tensile performance of concrete. Additionally, the dense distribution of aggregate particles forces cracks to circumvent the ITZ instead of directly penetrating the aggregates, thereby increasing the tortuosity and complexity of crack paths. This enhances the energy dissipation capability and prevents further reduction in peak strength.

**Figure 8:**
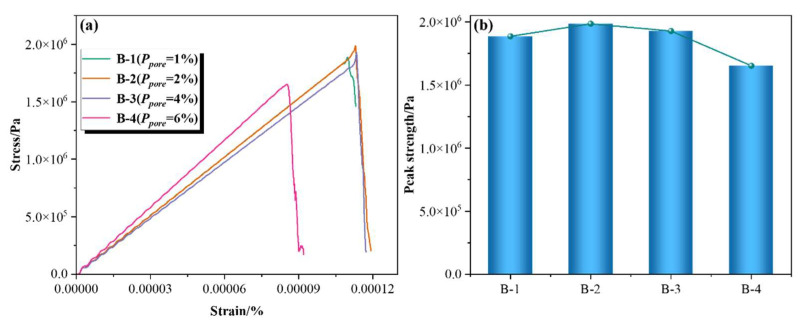
Variation of tensile strength of concrete with different pore percentages. (**a**) Stress–strain curve and (**b**) variation of peak strength.

Figure 8 presents the strength variation under different porosity percentages. It is observed that with increasing pore percentage *P_pore_*, the peak tensile strength initially increases and then decreases. When *P_pore_* increases from 1% to 2%, the pores act as minor initial defects, and their influence on peak strength is limited. Cracks still primarily initiate at weak contact points within the ITZ or mortar. As *P_pore_* increases to 4%, both the distribution density and connectivity of pores increase. Stress concentration around pore edges becomes the dominant mechanism for crack initiation, resulting in a reduction of approximately 3% in peak strength compared to the 2% case. When *P_pore_* further increases to 6%, the internal continuity of the concrete is severely compromised. The pores form an interconnected defect network, allowing cracks to propagate rapidly along connected pores. This results in a low-energy-dissipation failure mode and a 14% drop in peak strength compared to the previous case. This trend highlights the dual role of pores in weakening the effective load-bearing area and serving as crack-inducing defects. High porosity not only reduces the compactness of the mortar matrix but also accelerates microcrack initiation and coalescence through stress concentration, ultimately causing a marked deterioration in tensile strength.

The effects of aggregates and pores on peak tensile strength fundamentally stem from their distinct roles in the microstructure. Aggregates, as high-strength phases, influence strength by modifying the ITZ area and crack propagation paths, embodying a competitive relationship between the weak interface effect and the skeleton support effect. In contrast, pores, as defect phases, directly compromise matrix continuity and alter stress distribution, following a near-linear strength degradation pattern. Together, these findings reveal the microscopic correlation mechanism of “composition–defect–mechanical performance” in the multiphase heterogeneous structure of concrete, providing a theoretical foundation for improving tensile strength through aggregate gradation optimization and porosity control.

## 4. Discussion

### 4.1. Influencing Mechanisms of Microstructure on Crack Propagation

The cracking behavior of concrete is fundamentally the result of interactions among its multiphase components. As a high-strength phase, aggregate directly affects the area and distribution of the ITZ as its proportion varies: when the aggregate content increases from 30% to 40%, the ITZ area expands. Due to its lower mechanical strength compared to the mortar matrix, the ITZ becomes the preferred path for crack initiation and propagation, leading to a decrease in tensile strength. However, when the aggregate percentage further increases to 45%, the densely distributed aggregates form an effective load-bearing skeleton, forcing cracks to circumvent the ITZ rather than penetrate the aggregates. This increases the tortuosity of crack paths and enhances energy dissipation, thereby mitigating the continued strength reduction. The presence of pores influences the cracking process through a dual mechanism of stress concentration and reduction in effective load-bearing area. At low pore percentages, pores act as secondary defects, and cracks still primarily propagate along the ITZ. In contrast, at high pore percentages, increased pore connectivity leads to the formation of a defect network, promoting crack initiation at pore edges and rapid coalescence. This shifts the failure mode from ITZ-dominated to pore-dominated, revealing the regulatory role of defect phases in microscopic damage evolution.

Table 2 shows the comparison of the results of this study with previous studies, quantifying for the first time the nonlinear strength effect of aggregate percentage under tensile loading using DEM (turning point at 40%). And it was found that the failure mode transition threshold, when the pore percentage is greater than 4%, supplements the microscopic mechanism of defect evolution. This establishes a new benchmark for the application of DEM in concrete tensile analysis.

### 4.2. Advantages and Limitations of DEM Simulation

The Discrete Element Method directly models the heterogeneous structure of materials using discrete particle units, eliminating the need to predefine crack paths. This offers significant advantages in simulating discontinuous deformation processes such as crack initiation, propagation, and coalescence. In this study, DEM successfully simulated the progressive failure process of cracks propagating along the ITZ and mortar matrix. The simulated stress–strain curves showed strong agreement with experimental results, confirming the applicability of the parallel bond model (PBM) for characterizing the micromechanics behavior of concrete. However, the computational efficiency of DEM is constrained by the number of particles and the time step size. The current model has limitations: it does not account for aggregate angularity, microscale pore distribution in the mortar, or dynamic loading effects, which may affect the accuracy of real-world scenario simulations. Additionally, the 2D model may not fully capture the complex 3D crack networks in actual concrete structures. Future work may integrate CT imaging techniques to construct more realistic microstructures and extend the model toward multiscale coupled simulations.

### 4.3. Engineering Application and Optimization

Findings here offer quantitative guidance for high-performance concrete design. For bridge decks, the aggregate percentage should be maintained at 40–45%. In tunnel linings requiring high crack resistance, controlling porosity below 2% via curing optimization prevents strength collapse due to pore connectivity. In addition, DEM simulation can serve as a virtual testing tool to assist in optimizing concrete mix design and reduce the cost of physical experiments. It also offers technical support for long-term safety assessments in concrete engineering applications.

## 5. Conclusions

(1) A DEM-based numerical model incorporating mortar, aggregate, and the interfacial transition zone (ITZ) was developed in this study. The parallel bond model (PBM) was employed to simulate inter-particle mechanical behavior. By calibrating micromechanical parameters to match experimental tensile strength and failure modes, the model successfully reproduced the cracking mechanisms of concrete under tensile loading. These results demonstrate the effectiveness of DEM in investigating the cracking behavior of multiphase heterogeneous concrete structures. This study innovatively couples microstructural parameters (aggregate percentages and porosity) with macroscopic tensile properties, offering a new framework for meso-scale failure analysis.
(2) A series of simulation schemes with varying aggregate and pore percentages were designed to explore concrete behavior under tensile loading. The results reveal that damage primarily initiates and propagates within the mortar and ITZ regions. Aggregates, acting as a high-strength skeleton, force cracks to deflect along the ITZ rather than penetrate the aggregates, while pores act as defects that induce crack initiation and coalescence. This leads to the formation of a horizontal macro-scale tensile fracture zone.
(3) Tensile strength exhibits a non-linear response to aggregate content: a 26% decrease from 30% to 40% aggregate due to expanded ITZ weak interfaces, followed by a recovery at 45% due to the aggregate skeleton effect. While increasing pore percentage results in an opposite trend: 3% reduction at 4% pore strength and 14% at 6% pore strength, driven by defect connectivity.
(4) This study reveals the effects of percentages of aggregate and pore on concrete failure using DEM, providing tangible guidance for engineering practice: (1) optimizing aggregate gradation to balance ITZ area and skeleton support; (2) controlling porosity during construction to minimize initial defects; (3) using DEM simulations as a cost-effective tool for mix design and structural safety evaluation. The results are particularly applicable to engineering projects requiring high tensile durability, such as bridge decks, tunnel linings, and offshore structures.

## Figures and Tables

**Table 1:** Specific microscopic parameters.

Parameters of Aggregates		Parameters of Mortar	
Emod (Pa)	555 × 10^8^	Emod (Pa)	130 × 10^8^
Pb_emod (Pa)	555 × 10^8^	Pb_emod (Pa)	130 × 10^8^
Pb_ten (Pa)	20 × 10^6^	Pb_ten (Pa)	5 × 10^6^
Pb_coh (Pa)	25 × 10^6^	Pb_coh (Pa)	6 × 10^6^
Pb_fa (°)	40	Pb_fa (°)	45
Kratio	1.5	Kratio	2

**Table 2:** Comparison with previous studies.

Comparing Dimensions	Related Studies	Difference and Correlation Analysis
Research method	FEM simulation is the main approach, with most focusing on compression failure [37,38].	Traditional research often focuses on uniaxial compression, but this study achieves stretch simulation under bivariate control.
Influences of aggregate percentages	Zhao et al. [39] observed that an increase in aggregate percentage led to a monotonic increase in strength.	This study revealed through parameter calibration that 40% is the turning point between the ITZ weak interface effect and aggregate skeleton effect.
Influences of pore percentages	Li et al. [31] found that the strength decreases linearly with the increase in pore percentage.	Pore connectivity (rather than just percentage) is a key factor determining the failure mode.
Crack propagation mechanism	Nitka et al. [37] found that cracks propagate along the aggregate mortar interface, without quantifying the impact of ITZ.	This study quantified the competitive effects of ITZ area and pore connectivity on crack paths.

## Data Availability

The original contributions presented in this study are included in the article. Further inquiries can be directed to the corresponding authors.
